# Transcriptional Analysis Allows Genome Reannotation and Reveals that *Cryptococcus gattii* VGII Undergoes Nutrient Restriction during Infection

**DOI:** 10.3390/microorganisms5030049

**Published:** 2017-08-23

**Authors:** Patrícia Aline Gröhs Ferrareze, Rodrigo Silva Araujo Streit, Patricia Ribeiro dos Santos, Francine Melise dos Santos, Rita Maria Cunha de Almeida, Augusto Schrank, Livia Kmetzsch, Marilene Henning Vainstein, Charley Christian Staats

**Affiliations:** 1 Programa de Pós-Graduação em Biologia Celular e Molecular, Centro de Biotecnologia, Universidade Federal do Rio Grande do Sul (UFRGS), Porto Alegre 91501970, Brazil; p.ferrareze@outlook.com (P.A.G.F.); patriciaribsant@yahoo.com.br (P.R.d.S.); francinemelise@hotmail.com (F.M.d.S.); aschrank@cbiot.ufrgs.br (A.S.); liviak@cbiot.ufrgs.br (L.K.); 2 Departamento de Biologia Molecular e Biotecnologia, Instituto de Biociências, Universidade Federal do Rio Grande do Sul (UFRGS), Porto Alegre 91501970, Brazil; rodrigo.streit@hotmail.com; 3 Instituto de Física, Universidade Federal do Rio Grande do Sul (UFRGS), Porto Alegre 91501970, Brazil; rita@if.ufrgs.br

**Keywords:** *Cryptococcus gattii*, R265, transcriptome, genome, annotation, amino acid, bronchoalveolar lavage, cryptococcosis

## Abstract

*Cryptococcus gattii* is a human and animal pathogen that infects healthy hosts and caused the Pacific Northwest outbreak of cryptococcosis. The inhalation of infectious propagules can lead to internalization of cryptococcal cells by alveolar macrophages, a niche in which *C. gattii* cells can survive and proliferate. Although the nutrient composition of macrophages is relatively unknown, the high induction of amino acid transporter genes inside the phagosome indicates a preference for amino acid uptake instead of synthesis. However, the presence of countable errors in the R265 genome annotation indicates significant inhibition of transcriptomic analysis in this hypervirulent strain. Thus, we analyzed RNA-Seq data from in vivo and in vitro cultures of *C. gattii* R265 to perform the reannotation of the genome. In addition, based on in vivo transcriptomic data, we identified highly expressed genes and pathways of amino acid metabolism that would enable *C. gattii* to survive and proliferate in vivo. Importantly, we identified high expression in three APC amino acid transporters as well as the GABA permease. The use of amino acids as carbon and nitrogen sources, releasing ammonium and generating carbohydrate metabolism intermediaries, also explains the high expression of components of several degradative pathways, since glucose starvation is an important host defense mechanism.

## 1. Introduction

*Cryptococcus gattii*, along with its sibling species *Cryptococcus neoformans*, are the etiological agents of cryptococcosis, a life-threatening disease that particularly affects the lungs and central nervous system. Cryptococcosis is a major health problem on the African continent and other tropical regions, with an extremely high fatality ratio among immunocompromised patients [1] and is, therefore, considered one of the most prevalent fatal fungal diseases worldwide [2]. Although accountable for 20% of cryptococcosis cases [3], *C. gattii* is also capable of infecting immunocompetent patients [4]. In addition, the identification of hypervirulent strains in the outbreaks on Vancouver Island [5] and in the United States [6] has driven efforts to identify the genetic and molecular differences responsible for the increased virulence of *C. gattii* [4].

Cryptococcosis is initiated by the inhalation of infectious propagules (as spores and dried yeast cells). After reaching the alveolar parenchyma, yeast cells are typically phagocytosed by macrophages, within which *C. gattii* can survive and replicate [7]. In addition, cryptococcal cells are considered a facultative intracellular pathogen of neutrophils and dendritic cells, defense cells which act during the early stages of murine pulmonary infection [8]. These innate immune cells have antigen-presenting activity and produce proinflammatory cytokines, inducing an adaptive immune response in the host [8,9]. The capability of *Cryptococcus* cells to avoid phagocytosis, as well as to survive and replicate at a high rate inside phagocytes, contributes to the dissemination of fungal cells from the lung via the bloodstream [10,11]. Among all the virulence factors of *Cryptococcus*, the most important element for preventing phagocytosis is the capsule, as its immunosuppressive functions are directly involved in cryptococcal survival within phagocytic cells. Internalized cryptococcal cells can inhibit the acidification of phagosomes in macrophages, leading to phagosome-lysosome fusion membrane disruption [10,12]. Phagolysosomal membrane damage, in turn, enables *Cryptococcus* to escape the phagolysosome, thus accessing the nutrients in the cytoplasm [11].

The nutritional immunity concept refers to an innate immune activity characterized by limitation by the host of essential nutrients in order to inhibit pathogen survival and proliferation. In this context, metals such as iron, copper, and zinc, which are necessary cofactors for at least one-third of proteins, constitute an important target of this mechanism [13,14]. To adapt to the stringent conditions imposed by the host, *C. neoformans* triggers the expression of membrane transporters for hexoses, amino acids, fatty acids, metals, ammonium, and phosphate, as well as stress response genes [15,16]. Additionally, alternative carbon sources such as acetate, lactate, fatty acids, or amino acids are used as primary carbon sources by cryptococcal cells during interaction with host cells [15].

Thus, considering the nutrient limitation imposed on *C. neoformans* cells in the lungs and that the *C. gattii* VGII representative strain primarily causes pulmonary disease [17], we performed transcriptional analysis of *C. gattii* R265 strain, recovered from bronchoalveolar lavage of infected mice. Functional mapping of expressed genes revealed that several genes involved in the uptake and metabolism of nutrients displayed high expression, suggestive of adaptation to a limited availability of carbon and nitrogen sources.

## 2. Materials and Methods

### 2.1. Ethics Statement

The use of animals in this work was approved by the Universidade Federal do Rio Grande do Sul Ethics Committee for Use of Animals (CEUA—protocol number 18807, approved on 10 May 2011). Mice were housed in groups of four in filtered top-ventilated cages, with a 12 h dark/light cycle and food and water ad libitum. The animals were cared for according to the guidelines of the Brazilian National Council for Animal Experimentation Control (CONSEA) and the Brazilian College of Animal Experimentation (COBEA). All efforts were made to minimize animal suffering.

### 2.2. RNA-seq Data

For bronchoalveolar lavage (BAL), 30 BALB/c mice were anesthetized with 100 mg/kg ketamine and 16 mg/kg xylazine in phosphate-buffered saline (PBS; NaCl 137 mM; KCl 2.7 mM; Na_2_HPO_4_ 10 mM; KH_2_PO_4_ 8 mM; pH 7.4) and nasally infected with 1 × 10^6^ *C. gattii* R265 cells in PBS. After 24 h, bronchoalveolar lavage was performed via tracheal cannula using three consecutive lavages of 1 mL of PBS. Following centrifugation, the recovered cells were merged in a single biological sample and lysed by washing with cold ultrapure H_2_O. RNA extraction from recovered yeast cells was performed using glass beads and an RNeasy Mini kit according to the manufacturer instructions (Qiagen, Hilden, Germany). RNA integrity and concentration were assessed by electrophoresis on a 1% agarose gel and by fluorometric analysis using a Qubit fluorometer and a Quant-iT RNA assay kit according to the manufacturer’s instructions (Invitrogen, San Diego, CA, USA). mRNA was purified from total RNA, processed, and a single-end sequencing was performed using Solexa technology on an Illumina Genome Analyzer GAII (Fasteris Life Sciences SA, Plan-les-Ouates, Switzerland). After quality analysis employing FastQC [18], low quality bases were filtered using FastX-Toolkit [19].

The *C. gattii* R265 libraries of WT and *ZAP1* null mutant (both grown in zinc deprivation conditions) used in this study were obtained from Schneider and colleagues [20], available at the NCBI SRA databases under accession codes SRX2523180 and SRX2522699, respectively.

### 2.3. Reads Alignment and Gene Prediction Refinement

**Figure 1:**
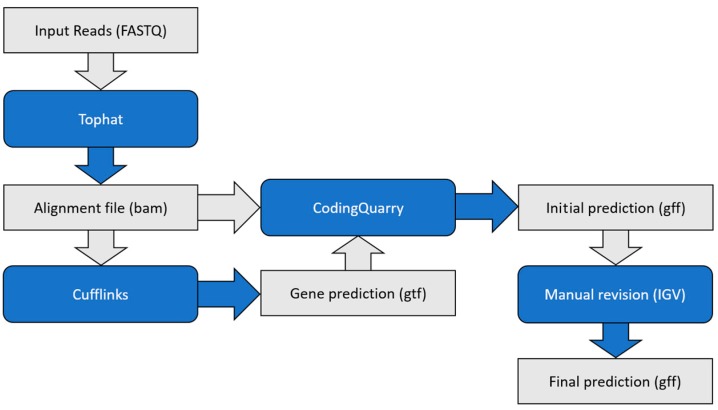
Gene prediction workflow. Reads were first aligned against the genome sequence using Tophat software. The alignment file (BAM) generated was then used to predict gene models (gtf) using Cufflinks software. Finally, both the alignment file and the gene models were loaded to CodingQuarry to generate the initial gene models, which are manually revised to generate a final prediction (gff).

The genome and transcript annotation of the R265 strain was downloaded from the Broad Institute archive [21] in August 2015. The reads were aligned against the genomic sequence using Tophat v2.0.13 software [22], with the following settings: minimum intron length, 10; maximum intron length, 5000; minimum segment intron, 10; and maximum segment intron, 5000. The alignment file was uploaded to Cufflinks v2.2.1 software [23] to generate the initial gene predictions, with the following settings: minimum intron length, 10; maximum intron length, 500; overlap radius, 10; and minimum isoform fraction, 0.4. Next, we performed CodingQuarry [24] predictions using the alignment file from Tophat and the gene predictions of Cufflinks to produce the preliminary transcriptome annotation. The alignment file, the new transcriptome annotation, and the transcriptome annotation from the Broad Institute were then uploaded to the Integrative Genomic Viewer software [25] for manual revision of both annotations. Exon/intron boundary predictions with less than 30 reads aligned, but those predicted in the previous annotation retained their structure as in the previous annotation. Exon/intron boundaries with no read support and no previous annotation were excluded. In addition, the untranslated regions (UTRs) were manually added for all predicted gene models based on the alignment. A fluxogram describing the gene prediction strategy is presented in Figure 1.

### 2.4. RNA Isolation and RT-PCR

*C. gattii* strain R265 cells were incubated in yeast peptone dextrose (YPD) media overnight at 200 rpm and 30 °C. Cells were then centrifuged (5000× *g* for 5 min) and washed in PBS. The cell pellet was suspended in 20 mL of yeast nitrogen base (YNB) and diluted to 1 × 10^6^ cells/mL. The cells were inoculated in 100 mL of YNB plus 10 µM of zinc chelator *N*,*N*,*N′*,*N′*-tetrakis(2-pyridylmethyl)ethane-1,2-diamine (TPEN) and incubated for 4 h at 200 rpm and 30 °C. The cells were collected by centrifugation (5000× *g* for 5 min), frozen in liquid nitrogen, and placed in an ultrafreezer (−80 °C) for lyophilization.

RNA isolation was performed using Trizol (Invitrogen–Life Technologies, Carlsbad, CA, USA) after cellular lysis via mortar and pestle. RNA integrity and quantification were assessed by electrophoresis on a 0.8% agarose gel and by spectrophotometry on a NanoDrop 2000 (Thermo Fisher Scientific, Wilmington, DE, USA). cDNA was prepared from DNAse-treated total RNA samples (1 µg) using Improm II Reverse Transcriptase (Promega, Madison, WI, USA) and oligo-dT. PCR was performed on a ProFLex PCR system (Applied Biosystems–Life Technologies, Carlsbad, CA, USA) with the following thermal cycling conditions: an initial step of 94 °C for 5 min followed by 30 cycles of 94 °C for 15 s, 55 °C for 15 s, and 72 °C for 60 s. All PCR products were subjected to agarose gel electrophoresis and visualized by staining with ethidium bromide.

### 2.5. BAL Expression Analysis

For expression analysis, the RNA-Seq library of *C. gattii* R265 from murine bronchoalveolar lavage was aligned against the R265 genome (NCBI accession code GCA_000149475.3) using Tophat [22] and the unique alignment option (max-multihits = 1). The expression values were measured by the fragments per kilobase per million (FPKM) using Cufflinks [23] and the proposed *C. gattii* R265 genome annotation.

### 2.6. Functinal Enrichment Analysis

For the detection of enriched functional KEGG and Gene Ontology terms, we selected genes classified as the most abundant according to the FPKM value distribution. Genes were collected in the FungiDB server [26], their orthologs from *C. gattii* WM276 identified in the same platform, and gene set enrichment analysis was conducted for the Biological Process and KEGG pathways. Only terms containing Benjamini-corrected *p*-values ≤ 0.05 were considered to be statistically enriched.

### 2.7. Transcriptogram

The transcriptogram was generated using the Transcriptogramer [27] program. For gene ordination, we used the STRING protein interaction data from *C. gattii* WM276 [28] with score ≥ 0.800. Expression analysis was performed using BAL FPKM expression data from Cufflinks. The enrichment analysis was evaluated using KEGG data for pathways and genes of *C. gattii* WM276 [29]. The conversion of WM276 genes to R265 was performed by a Blastp [30] orthologous search, with the best bidirectional hit model.

### 2.8. KEGG Pathway Mapping

For gene expression visualization within the phenylalanine, tyrosine, and tryptophan biosynthetic pathway, the Pathview server [31] was used. For KEGG pathway mapping, we used the WM276 gene names to R265 orthologs as well as the BAL expression values. The Pathview parameters were adjusted to a FPKM limit of 1000.

## 3. Results

### 3.1. Refinement of C. gattii R265 Genome Annotation

In order to understand the transcriptional profiling of *C. gattii* VGII on the site of infection, we performed RNA-Seq experiments to identify the most abundant transcripts. Purified mRNA samples from cryptococcal cells recovered from BAL fluid of BALB/c mice 24 h post infection were pooled and sequenced using the Solexa technology. After filtering low quality reads and/or bases, we obtained a total of 43,094,699 reads with a size of 100 nt. Our first attempt to align reads originating from the BAL library revealed that a consistent proportion of reads (81.41%) could not be aligned. In addition, we noted that only 65.04% of the reads aligned to genes, and 18.47% of the reads aligned to introns and intergenic regions. This led us to speculate that the current genomic annotation of *C. gattii* might contains errors, confirming our previous findings for the ZAP1 gene [20]. Therefore, we generated a new annotation based on transcriptomic evidence. Using our RNA-Seq dataset from three different growth conditions (*C. gattii* WT and ZIP1 null mutant exposed to a low zinc environment for 2 h, as well as cryptococcal cells recovered from BAL), we executed the CodingQuarry pipeline to generate new gene models for the R265 strain of *C. gattii*, which consisted of three steps (Figure 1). In the first step, all reads were aligned together against the genome of the R265 strain using Tophat software. In the second step, the alignment was loaded to Cufflinks software for an initial prediction. In the last step, both the predicted gene models of Cufflinks and the alignment from Tophat were loaded to the CodingQuarry software to obtain the refined gene models. Using this approach, we obtained 6956 protein coding gene models, which were then manually revised using IGV software.

During the manual revision, 509 gene models were excluded from the annotation due to a lack of alignment support or because their *open reading frames* did not show homology to any other fungal genes. Furthermore, 569 gene models predicted by the previous annotation but with a low count of reads spanning the intron-exon boundaries (less than 30 reads) had their previous annotation’s structure retained, as it was not possible to assure the accuracy of both prediction and revision. Finally, information on the 5′-UTR and 3′-UTR of the revised genes was manually added, as the prediction tools could not perform this step. After the revision, a total of 6411 gene models were proposed as the new transcriptome annotation (GFF File S1).

From the 6428 gene models comprising the annotation provided by the BROAD institute, we verified that only 3187 had their structure supported by the alignment data, meaning that more than a third of the gene models presented some type of error. Of these, 2563 genes contained misplaced intron-exon boundaries, sometimes leading the automated prediction program to exclude large extensions of the transcribed region in order to maintain the ORF that was initially predicted (Figure S1). In addition to the structural errors, 55 genes were excluded from the previous annotation. Two of these were excluded due to the identification of pseudogenes. The remaining 53 were excluded as a result of gene model fusion that incorrectly predicted separation in the last annotation. Thirty-eight potential new genes were also added, three of which were derived from the split of genes in the previous annotation that showed homology with genes from other strains of *C. gattii* and *C. neoformans*. In addition, we identified 257 punctual errors on the genome annotation of the strain R265 within the transcribed regions, which were associated with missing nucleotides, misplaced nucleotides, and wrong nucleotides (FASTA File S1).

### 3.2. Experimental Validation of the Gene Models

**Figure 2:**
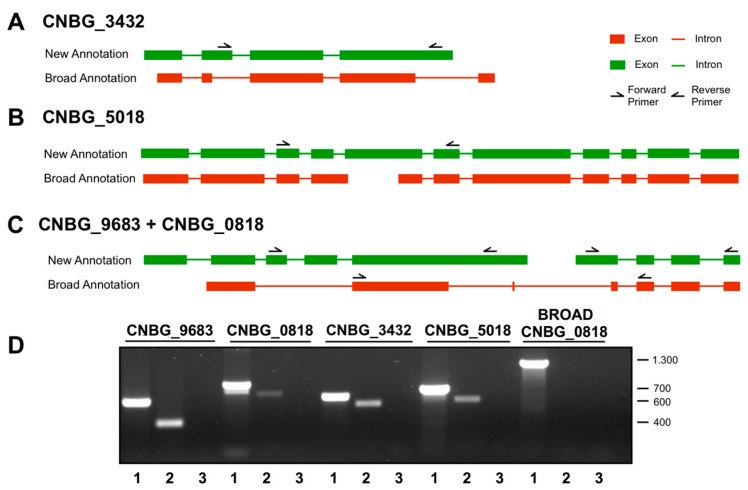
Experimental confirmation of the gene models. (**A**–**C**) Four genes were selected to confirm the changes in annotation provided by the RNA-Seq validation. Additionally, a primer pair designed to specifically amplify the predicted Broad annotation of the gene CNBG_0818 was design to confirm that it is in fact two genes. (**D**) The selected genes were confirmed by RT-PCR. For each gene, one genomic DNA (1) template, one cDNA template (2), and a negative control (3) were evaluated. All but Broad CNBG_0818 were amplified, indicating that the new annotation was correct. Values on the left refer to the band sizes (bp) of the molecular size marker. The green/red rectangles represent the exons, and the green/red lines between them are the introns; according the new and old (Broad) annotations, respectively.

In order to confirm the new annotation, we selected examples from the three major types of changes in the annotation: (i) genes that presented incorrectly identified intron-exon boundaries; (ii) genes that encompassed two split genes; and (iii) genes that encompassed one fused gene. Using the RNA-Seq data from the zinc limitation condition, we selected the most abundant gene in each group for confirmation, with the exception of group (iii), in which both genes comprising the gene with the highest expression in the previous annotation were selected. The genes selected for experimental validation were (i) CNBG_3432, (ii) CNBG_5018, and (iii) CNBG_0818 and CNBG_9683. RNA was isolated from cells cultured under the zinc limitation condition (YNB + 10 µM TPEN) for 2 h, and RT-PCR was used to confirm the new gene models (Figure 2).

### 3.3. Transcriptional Profiling of C. gattii Recovered from Murine BAL

**Figure 3:**
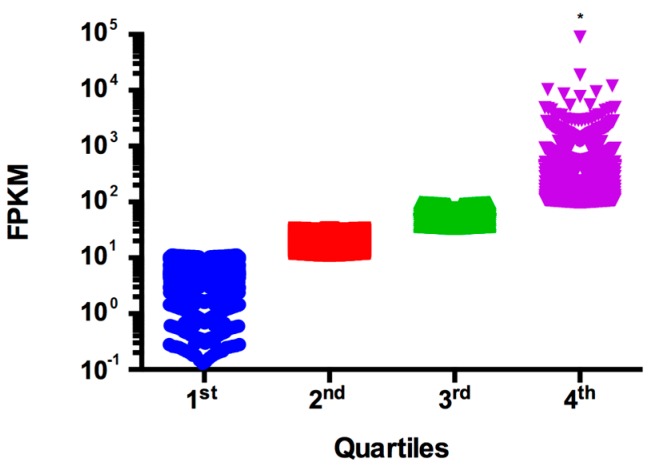
Transcriptional landscape of *C. gattii* R265 genes during pulmonary infection. FPKM values were determined from reads aligned to the *C. gattii* R265 genome using Cufflinks. FPKM values were distributed into quartiles and statistically analyzed using ANOVA followed by Tukey’s multiple comparisons test. The quartiles 1 (blue), 2 (red) and 3 (green) do not present statistical difference. The fourth quartile (purple) concentrates the abundant transcripts and has significant statistical difference (*) compared with others.

In order to determine RNA abundance in *C. gattii* cells at the site of infection, we recovered cryptococcal cells from mouse lung 24 h after infection. RNA was isolated from fungal cells and the sequences determined using Solexa technology. Measurements of transcript abundance using FPKM revealed that almost all genes could be detected. From the 6411 genes predicted in the *C. gattii* R265 genome, a total of 6257 presented FPKM values ≥ 1.0. However, the transcription levels of all genes ranged from 0 to 89,303.4. In addition, FPKM value distribution in quartiles revealed that 50% of transcripts had expression between 10 and 100 (quartiles 2 and 3), while the expression of the remaining genes (quartiles 1 and 4) ranged to two or three orders of magnitude, respectively (Figure 3 and Table S1).

### 3.4. Functional Profiling of C. gattii Transcriptome Recovered from Murine BAL

In order to functionally profile the most abundant genes of *C. gattii* recovered from BAL, we performed Gene Ontology enrichment using FungiDB [26] version 32. As the Gene Ontology annotation for *C. gattii* R265 is incomplete, we collected the IDs of *C. gattii* WM276 orthologs to most prevalent transcripts in the *C. gattii* R265 BAL library. The enriched terms refer mainly to processes involved in primary metabolism, such as those associated with synthesis and uptake of fundamental metabolites for cell survival (Table 1 and Table S2).

**Figure 4:**
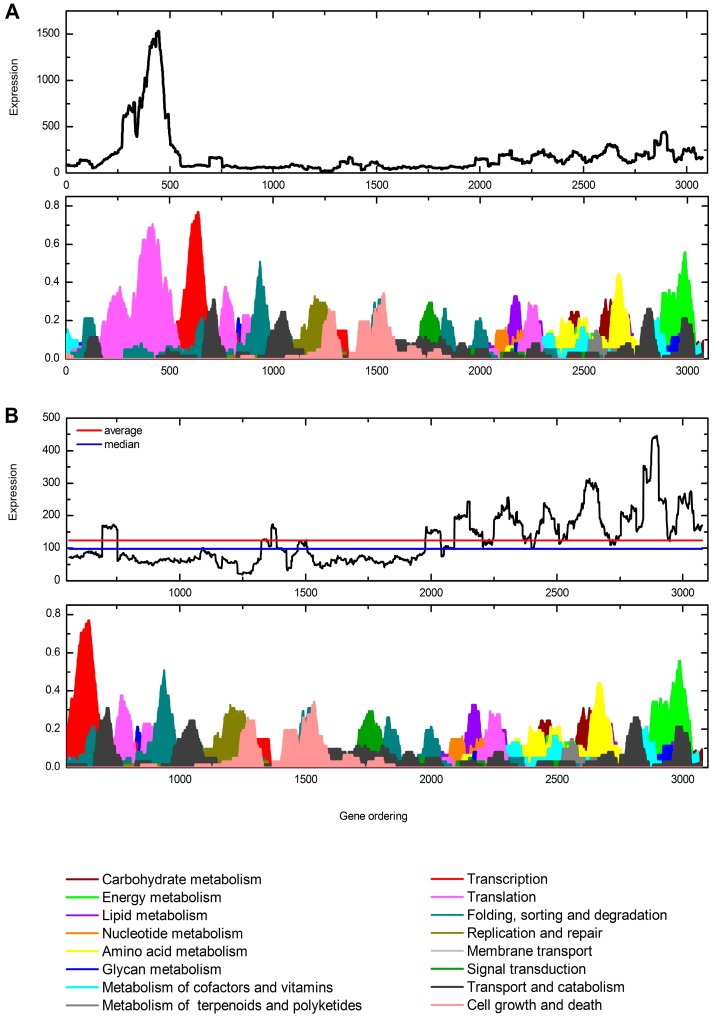
Transcriptogram of BAL RNA-Seq data of *C. gattii* R265. The upper graph shows the expression values (y) of ordered genes (x). The bottom graph indicates the KEGG pathways related to the ordered genes. (**A**) Complete transcriptogram; and (**B**) transcriptogram with the median and average expression values.

We expanded our functional profiling analysis using a transcriptogram [27], a tool that can measure the relative abundance of a given process for which a set of transcripts is assigned. The Transcriptogramer was used to take a snapshot of the metabolic pathways expressed during *C. gattii* infection. As the String and KEGG data were from *C. gattii* WM276, we collected information on orthologs from BLASTp analysis and generated an annotation file for *C. gattii* gene products based on their orthology with *C. gattii* WM276 gene products. The String networks provided information on protein interaction for 3076 genes (interaction score ≥ 0.800). We then obtained KEGG information for 2813 out of these 3076 genes for enrichment analysis (Figure 4A). As processes related to information processing (translation and ribosome biogenesis) displayed high expression values in the transcriptogram and this hinders the observation of enriched pathways, we determined the median of expression values from the genes associated to the remaining pathways. We then focused on the pathways whose associated gene expression was higher than the median (Figure 4B).

### 3.5. Virulence Gene Expression in C. gattii Recovered from Murine BAL

In order to evaluate the expression of virulence genes, we queried the pathogen–host interactions database (PHI-base) [32] using proteins coded by genes whose transcripts were defined as the most abundant in *C. gattii* recovered from BAL (FPKM ≥ 98.96). A total of 85 genes whose expression was classified as highly abundant were assigned as virulence factors based on this analysis (Table S3). The most abundant virulence factors were related to an extracellular elastinolytic metalloproteinase (CNBG_6001; FPKM = 4679.19) and a thiol-specific antioxidant protein 1 (CNBG_2132; FPKM 2709.54).

### 3.6. Nutrient Uptake Gene Expression in C. gattii Recovered from Murine BAL

A growing body of evidence suggests that cryptococcal cells undergo nutritional limitations in infection conditions and must, therefore, express nutrient uptake transporters on the cell surface in order to survive the harsh environment of the infection milieu [16,33,34,35]. Therefore, we determined the presence of transporter-coding transcripts among the most abundant genes. According to *C. gattii* R265 genome annotation, a total of 336 genes have the term “transporter” associated with at least one field in their annotation. Of these, 88 genes displayed FPKM values above 98.96, placing them as highly expressed genes (Table S4). The two most abundant transporters refer to a ribonucleotide transporter (ADP, ATP carrier protein; CNBG_4811; FPKM = 9360.59), and a glucose transporter (CNBG_0170; FPKM = 4905.2). However, transporters for other sugars, metals, and other compounds were found, suggesting that *C. gattii* responds to nutrient deprivation in infection conditions.

### 3.7. Nitrogen and Amino Acid Metabolism

Based on gene ontology enrichment analysis and transcriptogram profiling, we noted that amino acid metabolic pathways were enriched in cryptococcal cells recovered from BAL. This was confirmed by KEGG pathway enrichment analysis (Table S5), in which the following amino acid metabolic pathways appeared as enriched: cysteine and methionine metabolism (ec00270); glycine, serine, and threonine metabolism (ec00260); phenylalanine, tyrosine, and tryptophan biosynthesis (ec00400); alanine, aspartate, and glutamate metabolism (ec00250); valine, leucine, and isoleucine biosynthesis (ec00290); and valine, leucine, and isoleucine degradation (ec00280). This suggests that for some amino acids, the host imposes a nutrient deficient condition. Thus, we evaluated the expression of each gene associated with amino acid metabolic pathways to infer the transport and metabolism of nitrogen and amino acids. Several amino acids and organic and inorganic nitrogen uptake transporters were detected with high expression values (FPKM ≥ 98.96; Table 2).

In addition, we found that several genes related to amino acid metabolism were present in the most abundant transcripts in *C. gattii* recovered from BAL (Table S5). We focused on phenylalanine, tyrosine, and tryptophan biosynthesis genes, as these appear enriched in KEGG enrichment analysis. According to the KEGG database, 60 genes present in *C. gattii* WM276 could be assigned to this pathway. From these, a total of 56 orthologs and two lineage-specific genes were found in *C. gattii* R265 according to the FungiDB. The expression of these genes was detected in BAL (Figure S2). Despite some genes presenting low FPKM values, the majority (62%) had expression values above the median of all transcript values (FPKM = 34.22), suggesting that aromatic amino acids are likely synthesized during cryptococcal infection of lungs.

**Figure 5:**
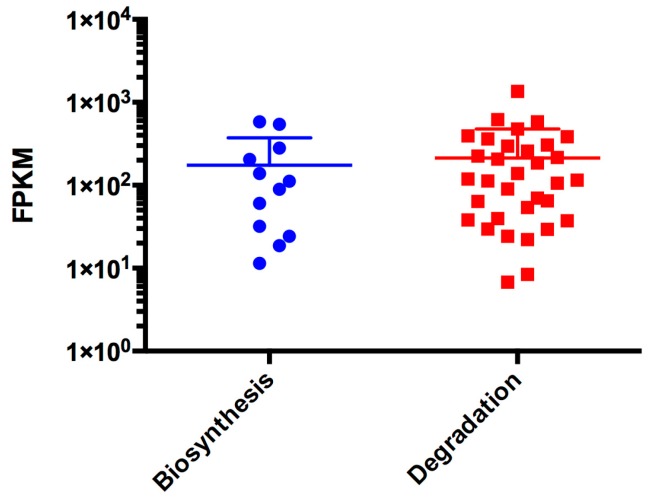
Transcriptional landscape of *C. gattii* R265 genes associated to valine, leucine, and isoleucine metabolism during pulmonary infection. FPKM values were determined from reads aligned to *C. gattii* R265 genome using Cufflinks software. Genes associated with biosynthesis (blues dots) and degradation (red squares) processes were recovered from The FungiDB.

We also investigated valine, leucine, and isoleucine metabolism, as both the biosynthesis and degradation of these amino acids appears as enriched among the most abundant transcripts in *C. gattii* cells recovered from BAL. For the biosynthesis pathway, 15 genes from *C. gattii* WM276 and *C. gattii* R265 were assigned based on the EC codes. For the degradation pathway, 44 genes present in *C. gattii* WM276 were assigned to this pathway. All the genes related to these pathways had their transcripts detected, suggesting that the corresponding amino acids are low in concentration during cryptococcal infection. However, as the mean FPKM values for valine, leucine, and isoleucine biosynthesis-associated genes were slight lower than those observed for their degradation (Figure 5), it is possible that a continuous process of synthesis and degradation takes place to generate acetyl-CoA during host adaptation as all of these pathways result in acetyl-CoA production.

The Gat1 transcription factor (CNBG_0368) is a specific nitrogen starvation regulator, and is expressed in BAL, despite in low levels (FPKM = 29.71). For means of comparisons, we determined the expression values of the *C. gattii* VGII orthologs (Table S6) to the *C. neoformans* transcription factors [36]. The median value of expression of *C. gattii* transcription factors is 20.25, which FPKM values ranging from 0 to 552.08 (Table S6). Along with the ammonium permease expression values, this may indicate that this condition is not subject to nitrogen starvation. Therefore, the high expression of GABA permease (CNBG_1602) indicates ammonium starvation and the utilization of secondary nitrogen sources, according to the expression of Ure2p. Moreover, *C. gattii* R265 showed high expression of some amino acid transporters (Table 2), suggesting amino acid uptake during host-pathogen interaction.

Taken together, the results suggest intense modulation of amino acid metabolism during the *C. gattii* R265 infection process.

## 4. Discussion

The task of describing the complete set of transcripts that an organism expresses can be extremely challenging and laborious. In some studies in which RNA-Seq is used to improve the transcript annotation, the corrections are performed manually, using the alignment as a guide [37]. Although this approach can improve the gene models and identify new genes, it demands a significant effort, especially where there are thousands of genes with incorrect structures. Therefore, we generated an entirely new set of transcripts using an alignment-based prediction tool, CodingQuarry. This program was designed to deal with the singularities of the fungal genome, such as the reduced intron size and the proximity of the genes, where other tools, such as Cufflinks, fail [24,37,38]. Although there was still a need for manual revision as not all the predicted models were correct, it was clear that a new prediction was more time-efficient than manually correcting thousands of genes.

With the final RNA-Seq based prediction, we corrected more than half of the transcripts predicted by the previous annotation and also identified 35 new genes. This result is similar to that obtained with transcriptome reannotation of the strain H99 of *C. neoformans* using RNA-Seq, in which almost 40% of the transcripts were changed and 55 new genes were found [39]. In contrast, other fungal species as *Candida albicans* and *Fusarium graminearum* showed less improvement with RNA-Seq-based correction [37,40]. This difference could be explained by the homology basis of automated annotation as, being basidiomycetes, *C. gattii* and *C. neoformans* are evolutionally more distant from the other well-described fungi. Therefore, we expect that the new transcript annotation of the *C. gattii* VGII R265 strain may allow the improvement of the annotation of other *C. gattii* strains. In addition, such gene models can be used by the cryptococcal research community. Public available platform, as the FungiDB, can be fed with the annotation of gene models and with transcriptome data to provide researchers fundamental information about expression of cryptococcal genes during the infection process. However, we are aware that we cannot refine the whole transcriptome. In this way, further experiments to profile the transcriptome would strengthen the reannotation and provide additional data for the correction of misannotated genes.

The new annotation, therefore, enabled new transcriptome analysis of *C. gattii* R265. We therefore evaluated some enriched pathways in these fungal cells obtained by bronchoalveolar lavage in a murine model of cryptococcosis. Although the nutrient composition of the phagosome is poorly defined, it is well accepted that it is a nutrient-poor environment [41]. Thus, several studies in *C. neoformans* have shown that such cells develop a strategy to cope with the scarcity of nutrients, since *S. cerevisiae* and *C. albicans* show that few, or no, amino acid biosynthesis genes are upregulated [15,42,43].

Nitrogen catabolite repression (NCR) is a mechanism that controls the utilization of optimal nitrogen sources in *Cryptococcus* species. In this context, NCR-regulated genes are repressed to ensure that secondary nitrogen source degrading pathways are not expressed when preferred nitrogen sources are available [44]. Thus, during nitrogen starvation, the expression of permeases and catabolic enzymes is activated by a specific GATA-factor family of transcription factors [45]. *S. cerevisiae* has two GATA factors involved in nitrogen metabolism, Gat1 and Gln3 [45]; *C. gattii* R265 has a Gat1 ortholog, the gene CNBG_0368 [46]. The utilization of amino acids and other nitrogenous sources requires their internalization by membrane permeases.

Ammonium uptake in *C. gattii* R265 is mediated by the low- and high-affinity ammonium permeases Amt1 and Amt2. Interestingly, the FPKM values of Amt2, which is transcriptionally induced in response to ammonium-limiting levels [47], does not indicate nitrogen deprivation under BAL conditions. However, Amt2 is also induced by low levels of alternative nitrogen sources [47], which might indicate that non-preferred nitrogen compounds, likely acquired by amino acid uptake and degradation, are supporting the minimum nitrogen requirements of the cell and avoiding Amt2 overexpression. Similarly, Ure2p (CNBG_2927) levels are low. In *S. cerevisiae,* the high intracellular concentration of Ure2p indicates nitrogen excess, and its inactivation by nitrogen limitation leads to NCR de-repression and GATA factor activation [48].

Another gene, the gamma-aminobutyric acid transporter, showed high expression in BAL. As observed by Luzzani and colleagues [49], GABA can induce UGA4 expression when cells are grown in nitrogen-poor conditions, but not when they are grown with ammonium, since GABA is used as a poor nitrogen source by *S. cerevisiae* [50] in an NCR-dependent mechanism.

*C. neoformans* expresses 10 genes encoding cytoplasm amino acid permease genes of the APC (amino acid–polyamine-choline transporter) superfamily: eight encode global permeases (AAP1 to AAP8) and two sulfur amino acid permeases (Mup1 and Mup3) with a high and low affinity for methionine and cysteine. The small number of amino acid permeases encoded by *Cryptococcus* genomes can be related to the low enzyme-substrate affinity [51]. Among the permeases, AAP3, AAP5, and AAP7 do not have true orthologues in R265. AAP6 (CNBG_6051), which displayed no transcriptional change according to nitrogen source (ammonium sulfate or amino acids) in *C. neoformans* studies [51], presented low levels. In *C. neoformans*, the deletion of AAP4 and AAP5 genes result in the highest impact on growth, indicating that AAP4 and AAP5 are highly redundant and essential for amino acid uptake, especially at 37 °C. Moreover, it was demonstrated that AAP4/AAP5 amino acid permeases are required as a virulence factor, since they participate in capsule production and stress resistance, and the aap4Δ/aap5Δ double mutant is avirulent in mouse and *Galleria mellonella* models. Despite the redundancy of AAP4/AAP5, *C. gattii* R265 appears to have just one ortholog to AA4 permease, CNBG_1371; this is consistent with the observation that just one is necessary for the thermal and oxidative stress response [51].

According to our analysis, some pathways related to amino acid biosynthesis were predicted to be enriched based on the FPKM of related genes from cryptococcal cells recovered from BAL. However, according to KEGG mapping, the enzymes involved in ammonium release by glutamate degradation are more abundant, suggesting that the in vivo condition prioritizes amino acid acquisition for degradation instead of synthesis. In addition, the production of acetyl-CoA from valine, leucine, isoleucine, and fatty acid degradation pathways, as well as from pyruvate and acetate by *Cryptococcus* during infection, is essential for the synthesis of chitin in the cell wall and O-acetylation of the capsule [52]. Similarly, all enriched degradative pathways that result in oxaloacetate formation contribute to gluconeogenesis [53].

The analysis of metabolites following co-incubation of *C. neoformans* with the lung epithelial cells of Liew and colleagues [52] showed that some compounds such as l-cysteine, lactic acid, pantothenic acid, fumaric acid, l-tyrosine, d-fructose, dl-3-phenyllactic acid, and 3-hydroxyisovaleric acid were potentially secreted by *C. neoformans* in supernatant culture media. In our study, we observed the enrichment of cysteine metabolism pathway in BAL, which could potentially explain the metabolic secretion during co-incubation.

In summary, we have generated a new annotation for the R265 strain of *C. gattii*, significantly improving the previous automated annotation. As this is the first RNA-Seq-based transcriptome annotation of a *C. gattii* strain, we believe that this annotation represents a valuable resource for the research community and will help to improve the annotation of other strains of *C. gattii*. Furthermore, all the results obtained in this work appear to correlate with amino acid uptake promoted by *Cryptococcus* cells in vivo. Furthermore, the accented degradation profiles confirm acquisition and degradation instead of the biosynthesis of nitrogen sources. The use of amino acids as carbon sources, generating carbohydrate metabolism intermediaries, also explains the high expression of many degradative pathways, since glucose starvation is an important host defense mechanism.

## Figures and Tables

**Table 1:** Gene ontology enrichment analysis of the most abundantly expressed genes in *C. gattii* recovered from BAL. The top ten enriched Biological Process terms are shown. Only Benjamini-corrected *p*-values < 0.5 were considered as significantly enriched.

ID	Name	Benjamini Corrected *p*-Value
GO:1901566	Organonitrogen compound biosynthetic process	8.59 × 10^−11^
GO:1901564	Organonitrogen compound metabolic process	3.73 × 10^−10^
GO:0006412	Translation	9.05 × 10^−10^
GO:0043043	Peptide biosynthetic process	1.53 × 10^−9^
GO:0043604	Amide biosynthetic process	2.40 × 10^−9^
GO:0006518	Peptide metabolic process	2.65 × 10^−9^
GO:0043603	Cellular amide metabolic process	6.16×10^−9^
GO:0044271	Cellular nitrogen compound biosynthetic process	4.24 × 10^−6^
GO:0055114	Oxidation-reduction process	5.89 × 10^−6^
GO:0008152	Metabolic process	6.56 × 10^−6^

**Table 2:** FPKM values of ammonium permeases, amino acid transporters, and GATA transcription factors.

GENE	DESCRIPTION	FPKM BAL
CNBG_0332	Ammonium permease 1 (AMT1)	415.458
CNBG_6023	Ammonium permease 2 (AMT2)	40.3613
CNBG_1602	Gamma-aminobutyric acid transporter	677.554
CNBG_3901	Gamma-aminobutyric acid transporter	268.427
CNBG_4571	Gamma-aminobutyric acid transporter	61.0116
CNBG_4665	Gamma-aminobutyric acid transporter	4.20125
CNBG_4156	Choline transporter	74.1659
CNBG_5513	l-methione transporter	11.9407
CNBG_4785	General amino acid transporter (AAP2)	441.806
CNBG_1371	General amino acid transporter (AAP4)	416.72
CNBG_9416	General amino acid transporter (AAP1)	363.575
CNBG_6051	General amino acid transporter (AAP6)	32.0951
CNBG_1350	Gamma-aminobutyric acid transporter (AAP8)	18.3341
CNBG_2012	Neutral amino acid permease	258.968
CNBG_1852	Neutral amino acid permease	194.189
CNBG_2927	Ure2p	21.9955
CNBG_4137	Bwc2	148.2437
CNBG_9614	Cir1	6.505
CNBG_0368	Gat1	29.7171
CNBG_3885	Gat201	340.642

## Supplementary Materials

The following are available online at www.mdpi.com/2076-2607/5/3/49/s1. **GFF File S1:** *C. gattii* R265 genome annotation—GFF file, **Figure S1:** Identification of annotation errors, **FASTA File S1:** Sequence file of *C. gattii* R265 genome annotation, **Table S1:** Cufflinks calculation of FPKM values from *C. gattii* R265 genes in BAL condition, **Table S2:** Statistical analysis of GO enrichment in the most abundant transcripts, **Table S3:** FPKM values of *C. gattii* R265 genes related to virulence, stress response, capsule, as well as mannoproteins, **Table S4**: FPKM values all identified transporters coding genes in *C. gattii* R265 genome, **Table S5:** FPKM values of amino acid metabolism related genes (biosynthesis and degradation), **Figure S2:** KEGG mapping of genes related to phenylalanine, tyrosine, and tryptophan biosynthesis, **Table S6:** FPKM values of predicted transcription factors.

## References

[1] Vallabhaneni S., Mody R.K., Walker T., Chiller T. (2016). The Global Burden of Fungal Diseases. Infect. Dis. Clin. N. Am..

[2] Rajasingham R., Smith R.M., Park B.J., Jarvis J.N., Govender N.P., Chiller T.M., Denning D.W., Loyse A., Boulware D.R. (2017). Global burden of disease of HIV-associated cryptococcal meningitis: An updated analysis. Lancet Infect. Dis..

[3] Meyer W., Gilgado F., Ngamskulrungroj P., Trilles L., Hagen F., Castañeda E., Boekhout T. (2011). Molecular Typing of the *Cryptococcus neoformans/Cryptococcus gattii* Species Complex. Cryptococcus.

[4] Chaturvedi V., Chaturvedi S. (2011). *Cryptococcus gattii*: A resurgent fungal pathogen. Trends Microbiol..

[5] Kidd S.E., Hagen F., Tscharke R.L., Huynh M., Bartlett K.H., Fyfe M., Macdougall L., Boekhout T., Kwon-Chung K.J., Meyer W. (2004). A rare genotype of *Cryptococcus gattii* caused the cryptococcosis outbreak on Vancouver Island (British Columbia, Canada). Proc. Natl. Acad. Sci. USA.

[6] Byrnes E.J., Marr K.A. (2011). The Outbreak of *Cryptococcus gattii* in Western North America: Epidemiology and Clinical Issues. Curr. Infect. Dis. Rep..

[7] Byrnes E.J., Li W., Lewit Y., Ma H., Voelz K., Ren P., Carter D.A., Chaturvedi V., Bildfell R.J., May R.C. (2010). Emergence and pathogenicity of highly virulent *Cryptococcus gattii* genotypes in the northwest United States. PLoS Pathog..

[8] Leopold Wager C.M., Hole C.R., Wozniak K.L., Wormley F.L. (2016). *Cryptococcus* and Phagocytes: Complex Interactions that Influence Disease Outcome. Front. Microbiol..

[9] Zhang M., Sun D., Shi M. (2015). Dancing cheek to cheek: *Cryptococcus neoformans* and phagocytes. Springerplus.

[10] DeLeon-Rodriguez C.M., Casadevall A. (2016). *Cryptococcus neoformans*: Tripping on acid in the phagolysosome. Front. Microbiol..

[11] Johnston S.A., May R.C. (2013). *Cryptococcus* interactions with macrophages: Evasion and manipulation of the phagosome by a fungal pathogen. Cell Microbiol..

[12] Tucker S.C., Casadevall A. (2002). Replication of *Cryptococcus neoformans* in macrophages is accompanied by phagosomal permeabilization and accumulation of vesicles containing polysaccharide in the cytoplasm. Proc. Natl. Acad. Sci. USA.

[13] Potrykus J., Ballou E.R., Childers D.S., Brown A.J. (2014). Conflicting interests in the pathogen-host tug of war: Fungal micronutrient scavenging versus mammalian nutritional immunity. PLoS Pathog..

[14] Kehl-Fie T.E., Chitayat S., Hood M.I., Damo S., Restrepo N., Garcia C., Munro K.A., Chazin W.J., Skaar E.P. (2011). Nutrient metal sequestration by calprotectin inhibits bacterial superoxide defense, enhancing neutrophil killing of Staphylococcus aureus. Cell Host Microbe.

[15] Hu G., Cheng P.Y., Sham A., Perfect J.R., Kronstad J.W. (2008). Metabolic adaptation in *Cryptococcus neoformans* during early murine pulmonary infection. Mol. Microbiol..

[16] Derengowski L.d.S., Paes H.C., Albuquerque P., Tavares A.H., Fernandes L., Silva-Pereira I., Casadevall A. (2013). The transcriptional response of *Cryptococcus neoformans* to ingestion by *Acanthamoeba*
*castellanii* and macrophages provides insights into the evolutionary adaptation to the mammalian host. Eukaryot. Cell.

[17] Ngamskulrungroj P., Chang Y., Sionov E., Kwon-Chung K.J. (2012). The primary target organ of *Cryptococcus gattii* is different from that of *Cryptococcus neoformans* in a murine model. MBio.

[18] Babraham Bioinformatics FastQC: A Quality Control Tool for High throughput Sequence Data. https://www.bioinformatics.babraham.ac.uk/projects/fastqc.

[19] Hannon Lab FASTX-Toolkit. http://hannonlab.cshl.edu/fastx_toolkit.

[20] De Oliveira Schneider R., de Souza Süffert Fogaça N., Kmetzsch L., Schrank A., Vainstein M.H., Staats C.C. (2012). Zap1 regulates zinc homeostasis and modulates virulence in Cryptococcus gattii. PLoS ONE.

[21] Broad Institute *Cryptococcus Gattii* R265 Genome and Annotation. http://archive.broadinstitute.org/ftp/pub/annotation/fungi/cryptococcus_gattii/genomes/cryptococcus_gattii_r265.

[22] Kim D., Pertea G., Trapnell C., Pimentel H., Kelley R., Salzberg S.L. (2013). TopHat2: Accurate alignment of transcriptomes in the presence of insertions, deletions and gene fusions. Genome Biol..

[23] Trapnell C., Roberts A., Goff L., Pertea G., Kim D., Kelley D.R., Pimentel H., Salzberg S.L., Rinn J.L., Pachter L. (2012). Differential gene and transcript expression analysis of RNA-seq experiments with TopHat and Cufflinks. Nat. Protoc..

[24] Testa A.C., Hane J.K., Ellwood S.R., Oliver R.P. (2015). CodingQuarry: Highly accurate hidden Markov model gene prediction in fungal genomes using RNA-seq transcripts. BMC Genom..

[25] Thorvaldsdóttir H., Robinson J.T., Mesirov J.P. (2013). Integrative Genomics Viewer (IGV): High-performance genomics data visualization and exploration. Brief. Bioinform..

[26] Stajich J.E., Harris T., Brunk B.P., Brestelli J., Fischer S., Harb O.S., Kissinger J.C., Li W., Nayak V., Pinney D.F. (2012). FungiDB: An integrated functional genomics database for fungi. Nucleic Acids Res..

[27] Rybarczyk-Filho J.L., Castro M.A.A., Dalmolin R.J.S., Moreira J.C.F., Brunnet L.G., De Almeida R.M.C. (2010). Towards a genome-wide transcriptogram: The *Saccharomyces cerevisiae* case. Nucleic Acids Res..

[28] String Database Protein Network Data from *Cryptococcus gattii* WM276. https://string-db.org/cgi/download.pl?UserId=6Sz4kwKfQN72&sessionId=DlfdC4uHXzBV&species_text=Cryptococcus+gattii+WM276.

[29] KEGG Pathways *Cryptococcus gattii* WM276. http://www.kegg.jp/kegg-bin/search_pathway_text?map=cgi&keyword=&mode=1&viewImage=true.

[30] Camacho C., Coulouris G., Avagyan V., Ma N., Papadopoulos J., Bealer K., Madden T.L. (2009). BLAST+: Architecture and applications. BMC Bioinform..

[31] Luo W., Pant G., Bhavnasi Y.K., Blanchard S.G., Brouwer C. (2017). Pathview Web: User friendly pathway visualization and data integration. Nucleic Acids Res..

[32] Urban M., Cuzick A., Rutherford K., Irvine A., Pedro H., Pant R., Sadanadan V., Khamari L., Billal S., Mohanty S. (2017). PHI-base: A new interface and further additions for the multi-species pathogen–host interactions database. Nucleic Acids Res..

[33] Dos Santos F.M., Piffer A.C., Schneider R.D.O., Ribeiro N.S., Garcia A.W.A., Schrank A., Kmetzsch L., Vainstein M.H., Staats C.C. (2017). Alterations of zinc homeostasis in response to *Cryptococcus neoformans* in a murine macrophage cell line. Future Microbiol..

[34] Jung W.H., Sham A., Lian T., Singh A., Kosman D.J., Kronstad J.W. (2008). Iron source preference and regulation of iron uptake in *Cryptococcus neoformans*. PLoS Pathog..

[35] Caza M., Kronstad J.W. (2013). Shared and distinct mechanisms of iron acquisition by bacterial and fungal pathogens of humans. Front. Cell. Infect. Microbiol..

[36] Jung K., Yang D., Maeng S., Lee K., So Y., Hong J., Choi J., Byun H., Kim H., Bang S. (2015). Systematic functional profiling of transcription factor networks in *Cryptococcus neoformans*. Nat. Commun..

[37] Zhao C., Waalwijk C., De Wit P.J.G.M., Tang D., Van Der Lee T. (2013). RNA-Seq analysis reveals new gene models and alternative splicing in the fungal pathogen *Fusarium graminearum*. BMC Genom..

[38] Schliebner I., Becher R., Hempel M., Deising H.B., Horbach R. (2014). New gene models and alternative splicing in the maize pathogen *Colletotrichum graminicola* revealed by RNA-Seq analysis. BMC. Genom..

[39] Janbon G., Ormerod K.L., Paulet D., Byrnes E.J., Yadav V., Chatterjee G., Mullapudi N., Hon C.C., Billmyre R.B., Brunel F. (2014). Analysis of the Genome and Transcriptome of *Cryptococcus neoformans* var. *grubii* Reveals Complex RNA Expression and Microevolution Leading to Virulence Attenuation. PLoS Genet..

[40] Bruno V.M., Wang Z., Marjani S.L., Euskirchen G.M., Martin J., Sherlock G., Snyder M. (2010). Comprehensive annotation of the transcriptome of the human fungal pathogen *Candida albicans* using RNA-seq. Genome Res..

[41] Haas A. (2007). The Phagosome: Compartment with a License to Kill. Traffic.

[42] Fan W., Kraus P.R., Boily M.J., Heitman J. (2005). *Cryptococcus neoformans* gene expression during murine macrophage infection. Eukaryot. Cell.

[43] Chen Y., Toffaletti D.L., Tenor J.L., Litvintseva A.P., Fang C., Mitchell T.G., McDonald T.R., Nielsen K., Boulware D.R., Bicanic T. (2014). The *Cryptococcus neoformans* transcriptome at the site of human meningitis. MBio.

[44] Russel Lee I., Chow E.W.L., Morrow C.A., Djordjevic J.T., Fraser J.A. (2011). Nitrogen metabolite repression of metabolism and virulence in the human fungal pathogen *Cryptococcus neoformans*. Genetics.

[45] Kmetzsch L., Staats C.C., Simon E., Fonseca F.L., Oliveira D.L., Joffe L.S., Rodrigues J., Lourenco R.F., Gomes S.L., Nimrichter L. (2011). The GATA-type transcriptional activator Gat1 regulates nitrogen uptake and metabolism in the human pathogen *Cryptococcus neoformans*. Fungal Genet. Biol..

[46] Ngamskulrungroj P., Chang Y., Roh J., Kwon-Chung K.J. (2012). Differences in nitrogen metabolism between *Cryptococcus neoformans* and *C. gattii*, the two etiologic agents of cryptococcosis. PLoS ONE.

[47] Rutherford J.C., Lin X., Nielsen K., Heitman J. (2008). Amt2 permease is required to induce ammonium-responsive invasive growth and mating in *Cryptococcus neoformans*. Eukaryot. Cell.

[48] Cunningham T.S., Andhare R., Cooper T.G. (2000). Nitrogen catabolite repression of DAL80 expression depends on the relative levels of Gat1p and Ure2p production in *Saccharomyces cerevisiae*. J. Biol. Chem..

[49] Luzzani C., Cardillo S.B., Moretti M.B., García S.C. (2007). New insights into the regulation of the Saccharomyces cerevisiae UGA54 gene: Two parallel pathways participate in carbon-regulated transcription. Microbiology.

[50] Cardillo S.B., Moretti M.B., García S.C. (2010). Uga3 and Uga35/Dal81 transcription factors regulate UGA4 transcription in response to γ-Aminobutyric acid and Leucine. Eukaryot. Cell.

[51] Martho K.F.C., De Melo A.T., Takahashi J.P.F., Guerra J.M., Da Silva Santos D.C., Purisco S.U., Melhem M.D.S.C., Dos Anjos Fazioli R., Phanord C., Sartorelli P. (2016). Amino acid permeases and virulence in *Cryptococcus neoformans*. PLoS ONE.

[52] Liew K.L., Jee J.M., Yap I., Yong P.V.C. (2016). In vitro analysis of metabolites secreted during infection of lung epithelial cells by *Cryptococcus neoformans*. PLoS ONE.

[53] Lorenz M.C., Bender J.A., Fink G.R. (2004). Transcriptional Response of Candida albicans upon Internalization by Macrophages Transcriptional Response of *Candida albicans* upon Internalization by Macrophages. Eukaryot. Cell.

