# Supplementary figures and images for: Transcriptional Analysis Allows Genome Reannotation and Reveals that Cryptococcus gattii VGII Undergoes Nutrient Restriction during Infection
Source: Microorganisms. 2017 Aug 23;5(3):49. doi: 10.3390/microorganisms5030049 (PMC5620640; doi:10.3390/microorganisms5030049)

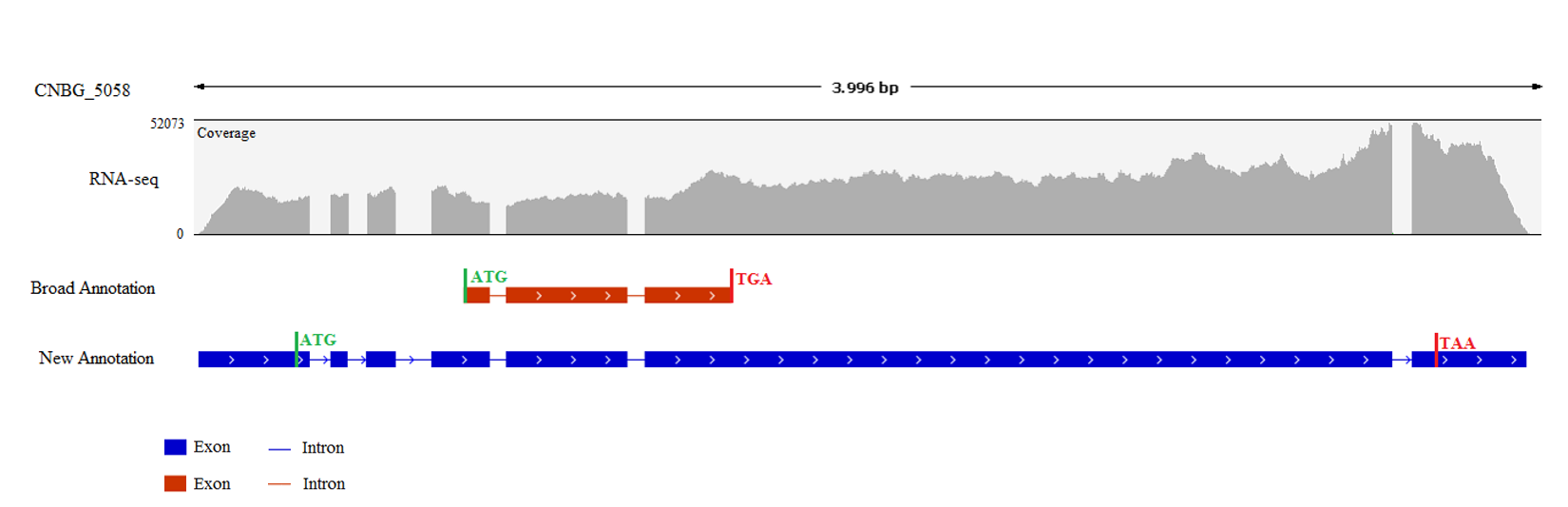

Supplement: Supplementary file 1 [file microorganisms-05-00049-s001.zip › Figure S1.tif]

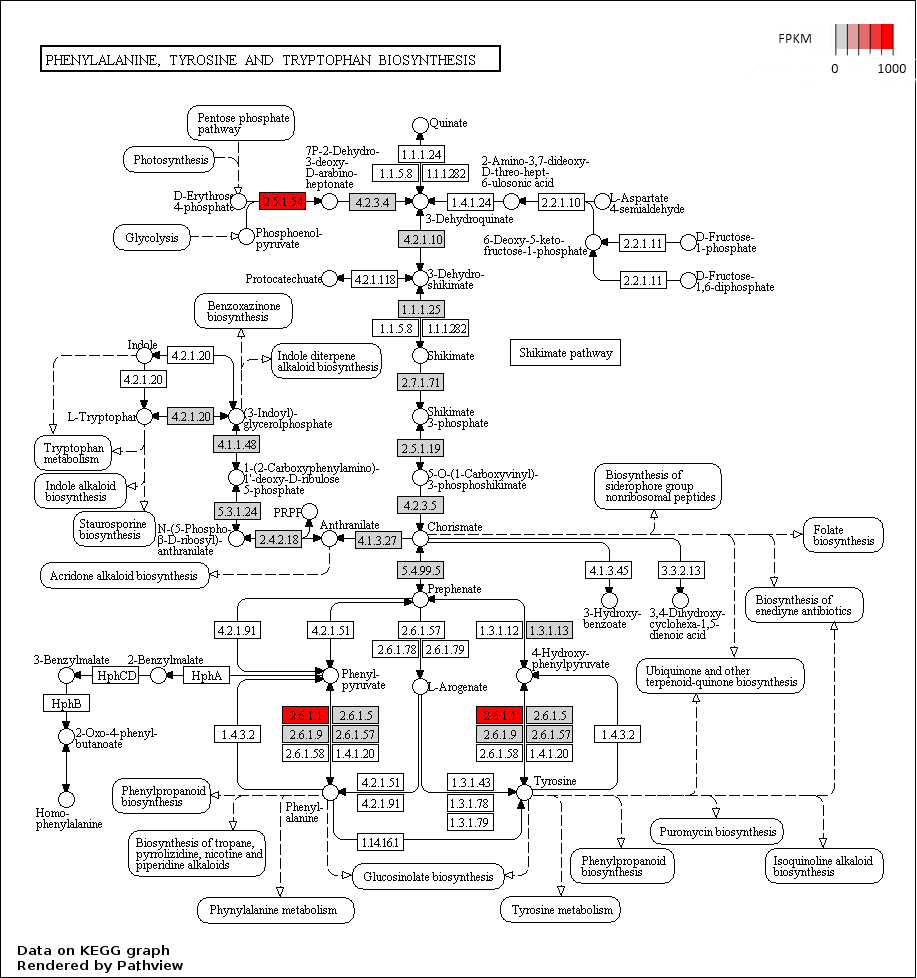

Supplement: Supplementary file 1 [file microorganisms-05-00049-s001.zip › Figure S2.png]
